# The *Leptin* Gene Family and Colorectal Cancer: Interaction with Smoking Behavior and Family History of Cancer

**DOI:** 10.1371/journal.pone.0060777

**Published:** 2013-04-08

**Authors:** Li Liu, Rong Zhong, Sheng Wei, Hao Xiang, Jigui Chen, Duoshuang Xie, Jieyun Yin, Li Zou, Jingwen Sun, Wei Chen, Xiaoping Miao, Shaofa Nie

**Affiliations:** 1 Department of Epidemiology and Biostatistics, and the Ministry of Education Key Lab of Environment and Health, School of Public Health, Tongji Medical College, Huazhong University of Science and Technology, Wuhan, Hubei, China; 2 Department of Epidemiology and Biostatistics, School of Public Health, Wuhan University, Wuhan, Hubei, China; 3 Department of Surgery, The Eighth Hospital of Wuhan, Wuhan, Hubei, China; 4 Department of Infection Control, Taihe Hospital, Yunyang Medical College, Shiyan, Hubei, China; The University of Texas M. D. Anderson Cancer Center, United States of America

## Abstract

**Background:**

Pathologic condition associated with metabolic syndrome traits seems to increase the risk of colorectal cancer. One mechanism underlying this relationship may involve the growth-promoting effects of the circulation hormones associated with obesity and insulin resistance, such as leptin.

**Methodology/Principal Findings:**

A two-stage case-control study was used to explore the role of polymorphisms of Leptin (*LEP*) and Leptin receptor (*LEPR*), either alone or in combination with environmental factors in colorectal carcinogenesis. In stage 1, 20 single nucleotide polymorphisms (SNPs) that tag common SNPs in these two genes were genotyped among 470 cases and 458 controls. In stage 2, another population with 314 cases and 355 controls were genotyped for the two most promising SNPs from stage 1. *LEPR* rs12037879 only presented modestly increased colorectal cancer risk, with odds ratios of 1.41 (95% confidence interval [CI] 1.13–1.76) and 1.74 (95%CI 1.08–2.81) for GA and AA genotype when compared with GG genotype in combined population. Smokers carrying *LEPR* rs12037879 A allele presented 1.67-fold (95%CI 1.39-fold to 2.01-fold) increased colorectal cancer risk when compared with non-smokers carrying GG genotype in combined analysis. Individuals with family history of cancer harboring *LEPR* rs12037879 A allele showed 1.52-fold (95%CI: 1.24-fold to 1.86-fold) increased colorectal cancer risk, compared with individuals without family history of cancer harboring GG genotype. Multifactor gene-environment interaction analysis revealed significant interactions among *LEPR* rs12037879, *LEPR* rs6690625, smoking status and family history of cancer, exhibiting a gradient of increased colorectal cancer risk along with the increasing number of risk factors (*P* = 9.82×10^−10^).

**Conclusions/Significance:**

Our research supports that polymorphisms in *LEPR* may be associated with marginal increase in the risk for colorectal cancer. Moreover, this association could be strengthened by cigarette smoking and family history of cancer.

## Introduction

Leptin, secreted primarily by adipocytes, is well-known to be crucial in energy balance, regulation of food uptake and nutrient absorption. In normal conditions, elevated leptin level could suppress food intake and promote the consumption of body fat by a negative feedback regulatory loop controlled by sympathetic nervous system. However, obese humans are resistant to the effects of endogenous leptin by a limitation of the blood-brain-barrier transport system for leptin and an inhibition of the leptin signaling pathway in leptin-responsive hypothalamic neurons [1]. Serum leptin level was viewed as a reflector of the amount of energy stored in the adipose tissues and in proportion to body fat mass, consequently, increased in the obese and decreased with weight reduction [2]. Consistently, lines of epidemiologic studies have demonstrated the connection between leptin and human obesity [3], [4].

Colorectal cancer is the second most frequently diagnosed cancer, with an estimated 1, 330, 000 new cases and 608, 000 cancer deaths in 2008 worldwide [5]. As complex disease, colorectal cancer has been long prevalent in western countries. During the past two decades, the incidence and mortality rates of colorectal cancer have grown rapidly in developing countries, including China [6]. Epidemiologic studies found that the incidence of colorectal cancer increased along with the rising of metabolic syndrome components, such as obesity [7]. Compared with normal weight, overweight or obesity presented 1.19-fold increased colorectal cancer risk [8]. Although, the underlying mechanisms remain unclear, previous evidence has suggested the involvement of abdominal visceral adipose tissue in the development of colorectal adenoma, the precursor of colorectal cancer [9]. Further evidence suggested that adipocytes and preadipocytes could exert growth stimulation role in colon cancer cells [10], and active substances produced by adipocytes could act as carcinogen in the colon and rectum [11].

Given the role of leptin in obesity development and the association between obesity and colorectal cancer risk, *Leptin* (*LEP*) was hypothesized as a contributor to colorectal cancer. The biological role of leptin was mediated through binding of specific cell surface receptors coupled to activation of PI3 kinase and Jak/Stat signaling, which exerts a critical role in the regulation of various cellular functions, including proliferation, differentiation and survival [12]. The leptin receptor was expressed in human colon cancer cell lines and human colonic tissue. Stimulation with leptin led to phosphorylation of p42/44 mitogen-activated protein kinase and increased cell proliferation in vitro and in vivo [13]. Significant decrease of tumor cell proliferation was observed in leptin-deficient tumors, and colon tumor growth was dramatically inhibited in leptin-deficient and leptin-receptor-deficient mice [14]. Recently, epidemiologic evidence has demonstrated the positive association between serum leptin and colorectal cancer risk. A case-control study conducted in Japan showed that, female with serum leptin level in quintile 2 and 3 combined, and quintile 4 and 5 combined harbored 1.40–fold and 4.84–fold increased colorectal cancer risk when compared with the lowest quintile, respectively [15]. Another nested case-control study conducted in Norway demonstrated that men with top quartile leptin level presented 2.28–fold increased colorectal cancer risk in comparison with three bottom quartiles [16]. A Chinese case-control study provided consistent evidence by finding an approximately two-fold increased risk of prostate cancer in men with the highest tertile leptin level relative to the lowest tertile [17].

Given the potential role of *Leptin* gene family in carcinogenesis and the influence of genetic polymorphisms in regulation of gene expression and function, it is inferred that polymorphisms in this gene family might exert an influence on cancer susceptibility. Previous study by Ribeiro et al. found that a functional polymorphism of *Leptin* (*LEP* -2548 G/A) increased susceptibility and earlier age of onset for non-small cell lung cancer [18]. Similarly, the tumorigenic role of *LEP* -2548G/A was also found in prostate cancer in American [19]. However, to date, these have been no studies addressing the role of genetic variants in *Leptin* gene family as colorectal cancer susceptibility factors in Chinese population. Therefore, we performed a two-stage case-control study to systemically evaluate single nucleotide polymorphisms (SNPs) of *LEP* and *LEPR* as a predictor of colorectal cancer risk in Chinese population.

## Materials and Methods

### Ethics Statement

Written informed consent referred to collection of individual’s demographic data including age, sex, weight and height, epidemiological information including smoking status, alcohol use, family history of cancer and physical activities, clinical data and blood left from clinical test was obtained from all final participants. This study protocol was approved by the review board of School of Public Health of Tongji Medical College of Huazhong University of Science and Technology in December, 2006 [No.200603].

### Study Participants

A two-stage case-control study design was utilized to estimate polymorphisms in *LEP* gene family in relation to colorectal cancer. Promising associations identified in stage I were validated in another study population. Study population in stage I and stage II came from Wuhan and Shiyan, respectively, which have been described previously [20]. Of eligible participants, 470 cases (94.0%) and 458 controls (91.6%) in first stage, and 314 cases (87.22%) and 355 controls (98.61%) in second stage completed in-person interviews, and donated blood samples, respectively. (Table S1).

### SNP Selection

A total of 119 SNP markers with a minor allele frequency (MAF)≥0.1 of *LEP* and *LEPR* were downloaded from HapMap (http://www.hapmap.org/) using phase 1 and phase 2 Data Release 24 (Build 36.3) for the Chinese population (Chinese Han from Beijing–CHB). Tag SNPs were chosen for each gene by using Tagger in Haploview (http://www.broadinstitute.org/haploview/haploview). We used the pair-wise mode and selected a minimal set of markers, such that all alleles to be captured would be correlated at an r^2^≥0.8 with a marker in that set [20]. Finally, *LEP* and *LEPR* yielded 4 and 16 tag SNPs, respectively. (Table S2).

### SNP Genotyping

Genomic DNA from peripheral blood samples were isolated using Blood Genomic DNA Purification kit (Tiangen Biotech, Beijing, China) following the manufacturer’s protocol.

The genotyping methods and quality control strategies in both stages have been described previously [20]. Briefly, in stage I, the genotyping assay was carried out through the Sequenom MassARRAY platform (Sequenom, San diego, CA, USA). The call rates of all SNPs were more than 96.5%. In stage II, two polymorphisms were genotyped using the 5′-nuclease (Taqman) assay (Applied Biosystems, Foster City, CA, USA). The call rates of both SNPs were more than 95.3%. In two stages, a total of 10% samples were genotyped in duplicate and showed 99.5% and 100% concordance, respectively.

### Statistical Analysis

Pearson’s χ^2^ test was used to compare the differences in distribution of categorical variables, and either Wilcoxon rank-sum test or Student’s *t*-test was used for continue variables, where appropriate. In this study, BMI was categorized as overweight or obese (BMI≥25 kg/m^2^) and non-overweight (BMI<25 kg/m^2^) [21]. Individuals who had smoked at least 100 cigarettes in their lifetimes were defined as smokers, and the rest were called non-smokers. Cumulative cigarette dose (pack-years) was calculated by the following formula: pack-years = [(number of cigarettes smoked per day) × (years smoked)]/20 cigarettes. Alcohol drinkers were defined as subjects who consumed at least 100 servings of any alcoholic beverages during their lifetime. [22] Hardy–Weinberg equilibrium was tested by a goodness-of-fit χ^2^ test to compare the observed genotype frequencies to the expected genotype frequencies in controls.

For the main effect of SNPs and two-way interactions, unconditional logistic regression was conducted to calculate odds ratios (ORs) and their corresponding 95% confidence intervals (CIs). The significance threshold under the Bonferroni correction for multiple testing was set at *P*<2.5×10^−3^ upon consideration of 20 SNPs analyzed.

To quantify the cumulative effect of gene-environment interactions, we tailed the total number of risk factors for each individual and set subjects without risk factors as the reference group. Colorectal cancer risk for individuals with different number of risk factors was estimated by calculating ORs and 95% CIs using unconditional logistic regression after adjustment for age and sex. All statistical analyses were two sided and performed using SPSS (SPSS, Inc., ver.12.0, Chicago, III., USA).

## Results

### Characteristics of the Study Population

Both of the two populations were from central China, in which, the colorectal cancer incidence ranked medially across the whole country, which might equal to the average level of 29.44/100000 in 2009 in China [23]. The exposure rates of potential cancer risk factors including overweight, smoking and drinking were 17.44%, 28.5% and 30.8% in average, respectively [24], [25]. The characteristics of our population were almost consistent with general population in central China (Table S1). There were no significant differences in the distribution of age and sex. The median age was 58 and 56 years old in controls in the first and second stage, respectively, compared with 58 and 59 years old in cases, respectively. 55.5% of cases and 56.3% of controls were male in the first stage, and 59.6% of cases and 58.6% of controls were male in the second stage. Alcohol use and BMI only presented marginal and modest influence on colorectal cancer risk in combined population. There were 30.2% self-reported alcohol users in cases, compared with 24.3% in controls of combined population. 25.5% and 20.4% were self-reported overweight or obesity in cases and controls, respectively, in combined population. Smoking status and family history of cancer presented different distribution between cases and controls. 37.4% and 28.5% were self-reported smokers in cases in the first and second stage, respectively, compared with 22.3% and 18.9% in controls (*P* for stage 1 = 5.86×10^−7^, *P* for stage 2 = 0.003). Given the significant role of smoking in colorectal carcinogenesis, we divided smokers into 3 groups (light smokers, medium smokers and heavy smokers) according to the tertile of pack-years of controls from combined population. Compared with non-smokers, medium smokers and heavy smokers presented higher colorectal cancer risk (OR = 1.69, 95%CI: 1.18–2.42 for medium smokers, and OR = 2.96, 95%CI = 2.13–4.12 for heavy smokers, respectively). In addition, more cases possessed family history of cancer in both stages (23.0% of cases vs 16.4% of controls in the first stage, *P* = 0.02; 13.1% of cases vs 5.4% of controls in the second stage, *P* = 0.001).

### Risk Associated with the Individual SNP

In the first stage, a total of 20 SNPs in *LEP* and *LEPR* were analyzed. All SNPs fit the Hardy-Weinberg equilibrium among controls. The distribution of genotypes of *LEPR* rs12037879 G/A was slightly different between cases and controls in both stages before Bonferroni correction. The frequencies of GG, GA and AA genotypes were 0.678, 0.283 and 0.039, and 0.685, 0.279 and 0.037 in controls in the first and second stage, compared with 0.604, 0.336 and 0.060, and 0.593, 0.349 and 0.058 in cases, respectively. In the first stage, the *LEPR* rs12037879 G/A polymorphism was associated with marginally increased colorectal cancer risk, with ORs of 1.34 (95%CI: 1.01–1.80) and 1.40 (95%CI: 1.06–1.85) for GA vs GG and GA+AA vs GG, respectively. In the validation study (Stage 2), the rs12037879 G/A polymorphism was consistently associated with modestly increased colorectal cancer risk, with ORs of 1.53 (95%CI: 1.09–2.14) and 1.57 (95%CI: 1.13–2.17) for GA vs GG and GA+AA vs GG, respectively. The increased colorectal risk was also observed in combined population with rs12037879 polymorphism no matter before or after Bonferroni correction. Individuals carrying rs12037879 GA genotype, AA genotype and A allele presented 1.41–fold (95%CI: 1.13–fold to 1.76–fold), 1.74–fold (95%CI: 1.08–fold to 2.81–fold) and 1.45–fold (95%CI: 1.18–fold to 1.79–fold) increased colorectal cancer risk compared with those carrying GG genotype, respectively (Table 1). None of other SNPs was found to play a role in colorectal cancer susceptibility whether after Bonferroni correction or not (Table S2 and S3). Given the importance of *leptin* gene family in the energy/body-mass mechanisms, we further detected whether rs12037879 correlated to any variation in the BMI of cases and controls respectively, to reveal the potential pathogenic mechanism of this polymorphism. However, rs12037879 showed no correlation with BMI either in cases or controls (Table S4).

**Table 1:** Potentially significant SNP associated with colorectal cancer risk.

Genotype	Stage 1			Stage 2			Combined Study		
*LEPR*rs12037879	No. (Case/Control)	OR(95%CI)^a^	*P* b	No. (Case/Control)	OR(95%CI)^a^	*P* b	No. (Case/Control)	OR(95%CI)^a^	*P* b
GG	282/309	1.00	0.05	185/243	1.00	0.04	467/552	1.00	0.002
GA	157/129	1.34(1.01–1.80)		109/99	1.53(1.09–2.14)		266/228	1.41(1.13–1.76)	
AA	28/18	1.85(0.98–3.51)		18/13	1.90(0.90–4.02)		46/31	1.74(1.08–2.81)	
GA+AA	185/147	1.40(1.06–1.85)		127/112	1.57(1.13–2.17)		312/259	1.45(1.18–1.79)	

a Adjusted by age, sex, smoking status and alcohol use.

b The cut-off point of *P* value was set as 2.5×10^−3^ under the Bonferroni correction for multiple testing.

### Two-way Interactions

Since gene-environment interactions always presented more important role in carcinogenesis than single genetic or environmental factor, we further explored potential interactions between SNPs in this gene family and environmental factors including smoking status, alcohol use, BMI and family history of cancer in colorectal cancer susceptibility. In the first stage, smokers carrying rs12037879 GA, AA genotype and A allele harbored 2.55–fold (95% CI: 1.55–fold to 4.19–fold), 5.27–fold (95% CI: 1.30–fold to 21.38–fold) and 2.93–fold (95% CI: 1.81–fold to 4.75–fold) increased colorectal cancer risk when compared with non-smokers carrying GG genotype. This interaction was also found in the second stage, during which, smokers harbored A allele showed increased colorectal cancer risk, with an OR of 1.47 (95% CI: 1.09–1.99), when compared with non-smokers harboring GG genotype. In the combined analysis, smokers carrying rs12037879 A allele presented 1.67–fold (95%CI: 1.39–fold to 2.01–fold) increased colorectal caner risk compared with non-smokers carrying GG genotype. *LEPR* rs12037879 also presented interaction with family history of cancer. Individuals with family history of cancer carrying A allele showed 2.49–fold (95%CI: 1.57–fold to 3.95–fold) and 1.52–fold (95%CI: 1.24–fold to 1.86–fold) increased colorectal cancer risk compared with individuals without family history of cancer carrying GG genotype in the second stage and combined population, respectively. Another *LEPR* polymorphism, rs6690625, although did not exert any significant main effect on cancer risk, but showed interactions with smoking status and family history of cancer in colorectal carcinogenesis (Table S2, S3 and S5). Compared with smokers carrying rs6690625 GG genotype, non-smokers carrying T allele presented decreased colorectal cancer risk, with ORs of 0.59 (95%CI: 0.42–0.81) and 0.69 (95%CI: 0.59–0.81) in the first stage and combined population, respectively. Individuals without family history of cancer carrying rs6690625 T allele also showed decreased colorectal cancer risk, with ORs of 0.61 (95%CI:0.44–0.82) and 0.74 (95%CI:0.63–0.87) in the first stage and combined population, respectively, compared with individuals with family history carrying GG genotype (Table 2). Neither *LEPR* rs12037879 nor rs6690625 presented significant interactions with BMI or alcohol use (Table S5).

**Table 2:** Two-way gene-environment interactions.

Variables	Stage 1		Stage 2		Combined study	
	OR(95%CI)^a^	*P*	OR(95%CI)^a^	*P*	OR(95%CI)^a^	*P*
*LEPR* rs12037879 × smoking status						
GG × never smoking	1.00		1.00		1.00	
GA× ever smoking	2.55(1.55–4.19)	2.21×10^−4^	1.51(0.80–2.88)	0.20	1.96(1.33–2.90)	0.001
AA × ever smoking	5.27(1.30–21.38)	0.02	2.41(0.58–9.97)	0.22	2.95(1.14–7.60)	0.03
(GA+AA) × ever smoking	2.93(1.81–4.75)	1.24×10^−5^	1.47(1.09–1.99)	0.01	1.67(1.39–2.01)	5.46×10^−8^
*LEPR* rs12037879 × family history of cancer						
GG × without family history of cancer	1.00		1.00		1.00	
GA × with family history of cancer	1.83(1.04–3.23)	0.04	6.27(1.78–22.09)	0.004	2.40(1.45–3.97)	0.001
AA × with family history of cancer	0.71(0.17–2.90)	0.63	/	/	1.15(0.35–3.80)	0.82
(GA+AA) × with family history of cancer	1.61(0.95–2.73)	0.08	2.49(1.57–3.95)	1.09×10^−4^	1.52(1.24–1.86)	4.75×10^−5^
*LEPR* rs6690625 ×smoking status						
GG × ever smoking	1.00		1.00		1.00	
GT× never smoking	0.55(0.39–0.77)	0.001	0.98(0.66–1.44)	0.90	0.73(0.57–0.94)	0.02
TT × never smoking	0.98(0.42–2.31)	0.97	0.99(0.40–2.44)	0.98	1.01(0.55–1.88)	0.97
(GT+TT) × never smoking	0.59(0.42–0.81)	0.001	1.24(0.81–1.91)	0.32	0.69(0.59–0.81)	4.91×10^−6^
*LEPR* rs6690625 × family history of cancer						
GG × with family history of cancer	1.00		1.00		1.00	
GT × without family history of cancer	0.59(0.41–0.83)	0.002	1.05(0.73–1.52)	0.79	0.76(0.59–0.72)	0.03
TT × without family history of cancer	0.82(0.34–1.94)	0.65	1.15(0.49–2.68)	0.75	0.98(0.54–1.79)	0.95
(GT+TT) × without family history of cancer	0.61(0.44–0.82)	0.003	0.79(0.61–1.05)	0.11	0.74(0.63–0.87)	2.76×10^−6^

a Adjusted by age, sex, smoking status and alcohol use.

### Combined Effect of Risk Factors

Since *LEPR* rs12037879, rs6690625 presented significant two-way interactions with smoking status and family history of cancer, we further detected multifactor interactions among these factors. We summed the number of risk factors of rs12037879, rs6690625, smoking status and family history of cancer for each individual and analyzed the resulting colorectal cancer risk in combined population. For environmental factors, smoking and with family history of cancer were chosen as risk factors. The genotypes of *LEPR* rs12037879 and rs6690625 were categorized as binary variables according to the potential risk tendency of each SNP in colorectal carcinogenesis exhibited previously (Table 1 and Table S2), namely rs12037879 GA or AA, and rs6690625 GG were viewed as risk factors. We found a significant dosage effect association for increased colorectal cancer risk with an increasing number of risk factors (*P*_for trend_ = 9.82×10^−10^). Compared with individuals without risk factors, individuals carrying 1, 2, 3 and 4 risk factors exhibited a gradient of increased colorectal cancer risk with adjusted ORs of 1.48 (95%CI: 1.07–2.06), 1.99 (95%CI: 1.42–2.77), 3.14 (95%CI: 2.06–4.79) and 7.60 (95%CI: 2.07–27.85), respectively (Table 3).

**Table 3:** Cumulative effect of risk factors of smoking, family history of cancer, *LEPR* rs12037879, and rs6690625 in colorectal cancer susceptibility in combined study.

No. of risk factors	No. (cases/controls)	OR(95%CI)^a^	*P*	*P*_ for trend_
0	74/137	1.00		9.82×10^−10^
1	253/318	1.48 (1.07–2.06)	0.019	
2	262/249	1.99(1.42–2.77)	5.43×10^−5^	
3	106/66	3.14(2.06–4.79)	1.10×10^−7^	
4	12/3	7.60(2.07–27.85)	0.002	

a Adjusted by age, sex.

## Discussion

This study systematically evaluated the association between a set of polymorphisms in the *Leptin* gene family and colorectal cancer risk in a two-stage case-control study. We found that *LEPR* rs12037879 was associated with a marginal increase in colorectal cancer risk. Moreover, *LEPR* rs12037879 and rs6690625 exhibited more important roles in colorectal carcinogenesis by interaction with smoking status and family history of cancer.

In the main-effect analysis, *LEPR* rs12037879 exhibited marginal association with increased colorectal cancer risk in combined population. This association might be biologically plausible. The *LEP* and its receptor gene were newly found to play a role in carcinogenesis especially in obesity-associated malignancies [26]. *Leptin* gene family existed roles in stimulation of DNA synthesis, enhancement of cell proliferation and survival promotion by regulating JAK-STAT, ERK1/2 and PI3K/AKT pathways. All the roles were helpful for the initiation of malignancy [27]. The gene family also induced angiogenesis by upregulating vascular endothelial growth factor [28] and promoted cell migration by the secretion of metalloproteinase [29]. Both functions mentioned above were crucial for tumor growth, invasion, and metastasis. Besides support from biologically functional evidence, elevated leptin concentration has been proven to promote the proliferation of colorectal epithelial cell by interacted with its receptor [13], whereas, LEPR-deficient mice presented increased susceptibility to azoxymethane-induced tumors [30]. Moreover, LEPR was significantly overexpressed in human colorectal cancer than normal colonic mucosa [31], and positively related with the expression of hypoxia-inducible factor 1, a proneoplastic transcriptional regulator, which caused a more advanced tumor phenotype [27]. Although, few epidemiologic studies have addressed the role of genetic polymorphisms of *LEP* gene family in colorectal cancer susceptibility systematically, previous evidence has indicated that SNPs in *LEPR* increased the risk for obesity and diabetes, which have been demonstrated as risk factors for various cancers [32]. Subsequently, variants in *LEPR* were also found to influence cancer risk in Caucasian, directly. For example, *LEPR* Q223R was found associated with an increased risk for oral squamous cell carcinoma [33], breast cancer [34] and non-small cell lung cancer [35]. Moreover, polymorphisms in intron 2 of *LEPR*, in which rs12037879 is located, have been demonstrated associated with basal-like beast cancer, which may also revealed the possibility of potential carcinogenesis role of genetic variants in this region [36].

Besides the modest main effect of *LEPR* rs12037879, we also observed significant gene-environment interactions, which were able to amplify the modest effect of the single genetic variant, and enhance the predictive power. In two-way interaction analyses, we found that *LEPR* rs12037879 presented significant interactions with smoking status and family history of cancer. Consistently, a significant factor-dosage effect was detected among *LEPR* rs12037879, *LEPR* rs6690625, smoking status and family history of cancer. The interaction between smoking and leptin family was first noticed in overweight study, during which, smoking was indicated, via nicotinic mechanisms, to modify the sensitivity of hypothalamic leptin receptor and consequently regulate leptin synthesis and reduce body weight [37]. Moreover, an epidemiologic study by Al Mutairi et al. [38] has shown that cigarette smoking presented significantly positive and dose-dependent correlation with leptin receptor in diabetic population. The interaction between cigarette exposure and leptin receptor has been further addressed in a study of chronic obstructive pulmonary disease, which inferred that underexpression of leptin receptor acted as a predisposing factor to cigarette smoking-induced lung disease [39]. Given the common pathogenesis between obesity, diabetes, COPD and cancer, and the individual carcinogenesis role of cigarette smoking and leptin gene family, we inferred a possible role of the interaction between smoking and *LEPR* polymorphism in colorectal cancer susceptibility. Although, little evidence has shown that family history of cancer exerted an influence on the expression of leptin and its receptor directly, previous studies have indicated that the expression of leptin gene family could be modified by family history of cancer-related diseases, such as diabetes [40]. Moreover, family history of cancer has been well-known to exert an influence on cancer susceptibility either alone or in combination with genetic variants long before [41]. Familial predisposition to cancer represented by individuals harbouring certain genetic defects, might strengthen the carcinogenesis role of *LEP* gene family, therefore, create a hot bed for tumor. In addition, the interaction between family history of cancer and *LEP* gene family has been addressed by Yapijakis et al., who found that compared to the controls, the homozygous high gene expression genotype AA of *LEP*-2548G/A was significantly increased in the subgroup of patients with positive family history of cancer [33]. As a SNP located in intronic region, rs6690625 did not present biological influence in gene expression or splicing, therefore, no main effect in carcinogenesis was found either in our study or previous researches. It might reflect insufficient pathogenicity of the single polymorphism. However, there was still evidence revealing that the SNP might have some indirect and tiny contribution to cancer susceptibility. For example, it has been reported to influence the age of menarche [42], which was indicated inversely related with colorectal cancer risk [43]. In addition, there were reports referring to the roles of SNPs captured by rs6690625 in cancer susceptibility, which might point out a potential and indirect correlation between rs6690625 and cancer risk. For instance, rs6588153, captured by rs6690625, possessed significantly elevated levels of acute-phase serum amyloid A [44], which was associated with various malignancies including colon cancer [45]. Moreover, rs6700896, also captured by rs6690625, presented strong association with C-reactive protein [46], a modest risk factor for colorectal cancer [47]. Given above background, it might reasonable that rs6690625 only presented a role in carcinogenesis in the existing of other risk factors, such as risk polymorphisms, cigarette smoking and family history of cancer.

There are some limitations in this study. First, both case-control studies were hospital-based, therefore, selection bias may exist, since the controls were from a health examination population which may not be ideal representatives of geographically matched population in similar environmental exposure. However, the controls came from the same region with cases and were randomly sampled, which may reduce the effect of selection bias. Second, the course of colorectal cancer is long and wasting, during which, many cancer patients may lose body weight due to the disease, therefore, retrospective BMI data in case-control study may not reflect the level of obesity before initiation of cancer. However, a large case-control study indicated that BMI based on recent self-reported measures reported similar result with BMI from prospective studies in colorectal cancer risk [48], [49], [50]. So, we infer recent self-reported BMI might bring substantial bias to the results, but not significant. Third, there are some missing data in environmental exposure in both case-control studies, such as family history of cancer, since the participants could not give exact information on related items during interviewing. Therefore, further studies with bigger sample size on gene-environment interactions are needed.

In conclusion, we provide evidence that one SNP in *LEPR* (rs12037879) may be associated with a marginal increase in colorectal cancer risk. Besides, *LEPR* rs12037879 present more important roles by interaction with smoking status, family history of cancer and *LEPR* rs6690625 in colorectal carcinogenesis. Further studies with large sample size are needed to certify our findings.

## Supporting Information

Table S1 Characteristics of including participants in two-stage case-control study.
(DOC)

Table S2 The association between SNPs in *LEP* gene family and colorectal cancer risk in stage 1.
(DOC)

Table S3 The association between *LEPR* rs6690625 and colorectal cancer risk in Stage 2.
(DOC)

Table S4 The influence of rs12037879 and rs660625 in BMI variation.
(DOC)

Table S5 Two-way gene-gene or gene-environment interactions.
(DOC)
