# Supplementary material for: The Leptin Gene Family and Colorectal Cancer: Interaction with Smoking Behavior and Family History of Cancer
Source: PLoS One. 2013 Apr 8;8(4):e60777. doi: 10.1371/journal.pone.0060777 (PMC3620466; doi:10.1371/journal.pone.0060777)
Supplement: Table S1 — Characteristics of including participants in two-stage case-control study. (DOC) [file pone.0060777.s001.doc]

Table S1 Characteristics of including participants in two-stage case-control study

|  | Stage 1 | | | Stage 2 | | | Combined study | | |
| --- | --- | --- | --- | --- | --- | --- | --- | --- | --- |
|  | Cases  (n=470) | Controls (n=458) | *P* | Cases  (n=314) | Control  (n=355) | *P* | Cases (n=784) | Controls  (n=813) | *P* |
| Age |  |  |  |  |  |  |  |  |  |
| Median | 58 | 58 | 0.72 | 59 | 56 | 0.06 | 58 | 57 | 0.10 |
| Sex |  |  |  |  |  |  |  |  |  |
| Male | 261(55.5%) | 258(56.3%) | 0.81 | 187(59.6%) | 208(58.6%) | 0.80 | 448(57.1%) | 466(57.3%) | 0.94 |
| Female | 209(44.5%) | 200(43.7%) |  | 127(40.4%) | 147(41.4%) |  | 336(42.9%) | 347(42.7%) |  |
| Smoking status |  |  |  |  |  |  |  |  |  |
| never | 294(62.6%) | 348(77.7%) | 5.86×10^-7^ | 223(71.5%) | 288(81.1%) | 0.003 | 517(66.1%) | 636(79.2%) | 4.87×10^-9^ |
| ever | 176(37.4%) | 100(22.3%) |  | 89(28.5%) | 67(18.9%) |  | 265(33.9%) | 167(20.8%) |  |
| Alcohol use |  |  |  |  |  |  |  |  |  |
| never | 310(66.0%) | 319(71.4%) | 0.08 | 235(75.6%) | 287(81.1%) | 0.08 | 545(69.8%) | 606(75.7%) | 0.01 |
| ever | 160(34.0%) | 128(28.6%) |  | 76(24.4%) | 67(18.9%) |  | 236(30.2%) | 195(24.3%) |  |
| BMI (kg/m^2)^ |  |  |  |  |  |  |  |  |  |
| <25 | 338(73.5%) | 348(78.7%) | 0.07 | 239(77.6%) | 279(80.6%) | 0.34 | 557(74.5%) | 627(79.6%) | 0.02 |
| ≥25 | 122(26.5%) | 94(21.3%) |  | 69(22.4%) | 67(19.4%) |  | 191(25.5%) | 161(20.4%) |  |
| Family history of cancer |  |  |  |  |  |  |  |  |  |
| Without | 324(77.0%) | 371(83.6%) | 0.02 | 266(86.9%) | 336(94.6%) | 0.001 | 590(81.2%) | 707(88.5%) | 6.22×10^-5^ |
| With | 97(23.0%) | 73(16.4%) |  | 40(13.1%) | 19(5.4%) |  | 137(18.8%) | 92(11.5%) |  |
