# Supplementary material for: The Leptin Gene Family and Colorectal Cancer: Interaction with Smoking Behavior and Family History of Cancer
Source: PLoS One. 2013 Apr 8;8(4):e60777. doi: 10.1371/journal.pone.0060777 (PMC3620466; doi:10.1371/journal.pone.0060777)
Supplement: Table S2 — The association between SNPs in LEP gene family and colorectal cancer risk in stage 1. (DOC) [file pone.0060777.s002.doc]

Table S2 The association between SNPs in *LEP* gene family and colorectal cancer risk in stage 1

| Gene | Genotype | No. (Case/Control) | OR(95%CI) ^a^ | *P*^b^ |
| --- | --- | --- | --- | --- |
| *LEP* | rs12706832 |  |  | 0.91 |
|  | GG | 255/251 | 1.00 |  |
|  | GA | 180/178 | 0.98(0.74-1.29) |  |
|  | AA | 33/29 | 1.11(0.65-1.90) |  |
| *LEP* | rs11763517 |  |  | 0.51 |
|  | TT | 281/277 | 1.00 |  |
|  | TC | 159/162 | 0.93(0.70-1.24) |  |
|  | CC | 27/19 | 1.46(0.78-2.73) |  |
| *LEP* | rs11761556 |  |  | 0.85 |
|  | AA | 253/239 | 1.00 |  |
|  | AC | 180/181 | 0.92(0.70-1.22) |  |
|  | CC | 34/36 | 0.85(0.51-1.41) |  |
| *LEP* | rs2071045 |  |  | 0.49 |
|  | CC | 147/143 | 1.00 |  |
|  | CT | 238/216 | 1.09(0.81-1.48) |  |
|  | TT | 81/91 | 0.86(0.58-1.27) |  |
| *LEPR* | rs6657632 |  |  | 0.08 |
|  | GG | 344/318 | 0.75(0.55-1.01) |  |
|  | GA | 108/131 | 1.77(0.74-4.24) |  |
|  | AA | 16/9 |  |  |
| *LEPR* | rs1475398 |  |  | 0.21 |
|  | CC | 362/344 | 1.00 |  |
|  | CG | 93/105 | 0.88(0.63-1.21) |  |
|  | GG | 15/8 | 2.16(0.81-5.74) |  |
| *LEPR* | rs10736402 |  |  | 0.22 |
|  | TT | 381/355 | 1.00 |  |
|  | TC | 80/97 | 0.77(0.55-1.09) |  |
|  | CC | 9/6 | 1.39(0.48-4.00) |  |
| *LEPR* | rs6690625 |  |  | 0.07 |
|  | GG | 336/300 | 1.00 |  |
|  | GT | 117/145 | 0.73(0.54-0.99) |  |
|  | TT | 16/13 | 1.08(0.50-2.32) |  |
| *LEPR* | rs7534511 |  |  | 0.61 |
|  | AA | 332/308 | 1.00 |  |
|  | AG | 124/131 | 0.93(0.69-1.25) |  |
|  | GG | 14/16 | 0.83(0.39-1.76) |  |
| *LEPR* | rs12040007 |  |  | 0.17 |
|  | AA | 312/280 | 1.00 |  |
|  | AG | 134/157 | 0.77(0.57-1.02) |  |
|  | GG | 23/20 | 1.08(0.56-2.07) |  |
| *LEPR* | rs9436746 |  |  | 0.60 |
|  | AA | 347/328 | 1.00 |  |
|  | AC | 113/115 | 0.99(0.73-1.35) |  |
|  | CC | 10/14 | 0.72(0.31-1.67) |  |
| *LEPR* | rs1327118 |  |  | 0.18 |
|  | CC | 345/337 | 1.00 |  |
|  | CG | 113/106 | 1.11(0.81-1.52) |  |
|  | GG | 6/14 | 0.40(0.15-1.09) |  |
| *LEPR* | rs12037879 |  |  | 0.05 |
|  | GG | 282/309 | 1.00 |  |
|  | GA | 157/129 | 1.34(1.01-1.80) |  |
|  | AA | 28/18 | 1.85(0.98-3.51) |  |
| *LEPR* | rs9436740 |  |  | 0.22 |
|  | AA | 326/310 | 1.00 |  |
|  | AT | 132/125 | 0.99(0.74-1.34) |  |
|  | TT | 11/20 | 0.48(0.22-1.04) |  |
| *LEPR* | rs10749754 |  |  | 0.46 |
|  | AA | 370/346 | 1.00 |  |
|  | AG | 90/100 | 0.84(0.60-1.17) |  |
|  | GG | 10/7 | 1.35(0.50-3.64) |  |
| *LEPR* | rs13306519 |  |  | 0.44 |
|  | CC | 304/310 | 1.00 |  |
|  | CG | 142/133 | 1.11(0.83-1.48) |  |
|  | GG | 21/14 | 1.61(0.79-3.28) |  |
| *LEPR* | rs7555955 |  |  | 0.60 |
|  | AA | 317/297 | 1.00 |  |
|  | AG | 131/142 | 0.92(0.69-1.23) |  |
|  | GG | 18/17 | 1.02(0.50-2.08) |  |
| *LEPR* | rs12029311 |  |  | 0.96 |
|  | GG | 265/276 | 1.00 |  |
|  | GA | 156/161 | 0.92(0.70-1.23) |  |
|  | AA | 22/21 | 1.04(0.55-1.95) |  |
| *LEPR* | rs1782763 |  |  | 0.88 |
|  | CC | 353/362 | 1.00 |  |
|  | CT | 84/91 | 0.94(0.67-1.33) |  |
|  | TT | 5/4 | 1.22(0.32-4.67) |  |
| *LEPR* | rs3806318 |  |  | 0.47 |
|  | AA | 344/311 |  |  |
|  | AG | 108/136 | 0.83(0.59-1.16) |  |
|  | GG | 18/11 | 1.67(0.75-3.73) |  |

^a^ Adjusted by age, sex, smoking status and alcohol use

^b^ The cut-off point of *P* value was set as 2.5×10^-3^ under the Bonferroni correction for multiple testing
