# Supplementary material for: The Leptin Gene Family and Colorectal Cancer: Interaction with Smoking Behavior and Family History of Cancer
Source: PLoS One. 2013 Apr 8;8(4):e60777. doi: 10.1371/journal.pone.0060777 (PMC3620466; doi:10.1371/journal.pone.0060777)
Supplement: Table S3 — The association between LEPR rs6690625 and colorectal cancer risk in Stage 2. (DOC) [file pone.0060777.s003.doc]

Table S3 The association between *LEPR* rs6690625 and colorectal cancer risk in Stage 2

| Gene | SNP | Genotype | No. (Case/Control) | OR(95%CI) ^a^ | *P*^b^ |
| --- | --- | --- | --- | --- | --- |
| *LEPR* | rs6690625 | GG | 193/237 | 1.00 | 0.41 |
|  |  | GT | 93/91 | 1.23(0.87-1.76) |  |
|  |  | TT | 12/12 | 1.30(0.57-2.98) |  |

^a^ Adjusted by age, sex, smoking status and alcohol use

^b^ The cut-off point of *P* value was set as 2.5×10^-3^ under the Bonferroni correction for multiple testing
