# Supplementary material for: The Leptin Gene Family and Colorectal Cancer: Interaction with Smoking Behavior and Family History of Cancer
Source: PLoS One. 2013 Apr 8;8(4):e60777. doi: 10.1371/journal.pone.0060777 (PMC3620466; doi:10.1371/journal.pone.0060777)
Supplement: Table S4 — The influence of rs12037879 and rs660625 in BMI variation. (DOC) [file pone.0060777.s004.doc]

Table S4 The influence of rs12037879 and rs660625 in BMI variation

| SNPs | Cases |  | Controls |  |
| --- | --- | --- | --- | --- |
|  | No.(BMI≥25/ BMI<25) | OR(95%CI) ^a^ | No.(BMI≥25/ BMI<25) | OR(95%CI) ^a^ |
| *LEPR* rs12037879 |  |  |  |  |
| GG | 127/316 | 1.00 | 111/421 | 1.00 |
| GA | 54/203 | 0.67(0.48-1.02) | 42/179 | 0.90(0.62-1.34) |
| AA | 9/37 | 0.62(0.30-1.32) | 8/26 | 1.19(0.53-2.67) |
| *LEPR* rs6690625 |  |  |  |  |
| GG | 127/394 | 1.00 | 106/415 | 1.00 |
| GT | 58/140 | 1.27(0.86-1.84) | 49/191 | 1.02(0.69-1.48) |
| TT | 5/22 | 0.72(0.28-1.91) | 6/19 | 1.22(0.50-3.19) |

^a^ Adjusted by age, sex, smoking status and alcohol use
