# Supplementary material for: The Leptin Gene Family and Colorectal Cancer: Interaction with Smoking Behavior and Family History of Cancer
Source: PLoS One. 2013 Apr 8;8(4):e60777. doi: 10.1371/journal.pone.0060777 (PMC3620466; doi:10.1371/journal.pone.0060777)
Supplement: Table S5 — Two-way gene-gene or gene-environment interactions. (DOC) [file pone.0060777.s005.doc]

Table S5 Two-way gene-gene or gene-environment interactions

| Variables | Stage 1 | | Stage 2 | | Combined study | |
| --- | --- | --- | --- | --- | --- | --- |
| *LEPR* rs12037879 × *LEPR* rs6690625 | OR(95%CI) ^a^ | *P* | OR(95%CI) ^a^ | *P* | OR(95%CI) ^a^ | *P* |
| GG × TT | 1.00 |  | 1.00 |  | 1.00 |  |
| GA × GT | 1.16(0.69-1.96) | 0.57 | 1.88(1.05-3.37) | 0.03 | 1.43(0.97-2.10) | 0.07 |
| GA × GG | 1.43(1.04-1.96) | 0.03 | 1.33(0.89-1.98) | 0.16 | 1.40(1.09-1.79) | 0.01 |
| AA × GT | 0.43(0.08-2.28) | 0.32 | 1.24(0.30-5.14) | 0.76 | 0.74(0.26-2.13) | 0.58 |
| AA × GG | 2.41(1.18-4.91) | 0.02 | 2.18(0.91-5.19) | 0.08 | 2.26(1.31-3.91) | 0.004 |
| *LEPR* rs12037879 × drinking status |  |  |  |  |  |  |
| GG × never drinking | 1.00 |  | 1.00 |  | 1.00 |  |
| GA × ever drinking | 0.80(0.48-1.30) | 0.36 | 1.62(0.81-3.24) | 0.17 | 1.26(0.85-1.86) | 0.24 |
| AA× ever drinking | 1.22(0.66-2.27) | 0.52 | 4.61(0.52-40.77) | 0.17 | 1.61(0.68-3.86) | 0.28 |
| (GA+AA) ×ever drinking | 0.84(0.54-1.33) | 0.47 | 1.18(0.87-1.60) | 0.30 | 1.03(0.86-1.23) | 0.78 |
| *LEPR* rs12037879 × BMI |  |  |  |  |  |  |
| GG × (BMI<25kg/m^2^) | 1.00 |  | 1.00 |  | 1.00 |  |
| GA × (BMI≥25kg/m^2^) | 1.31(0.72-2.39) | 0.38 | 1.36(0.66-2.81) | 0.41 | 1.29(0.81-2.04) | 0.28 |
| AA × (BMI≥25kg/m^2^) | 1.17(0.29-4.69) | 0.82 | 1.70(0.28-10.38) | 0.57 | 1.26(0.43-3.68) | 0.67 |
| (GA+AA) ×(BMI≥25kg/m^2^) | 1.29(0.74-2.25) | 0.38 | 1.17(0.88-1.56) | 0.28 | 1.14(0.95-1.37) | 0.17 |
| *LEPR* rs6690625 × drinking status |  |  |  |  |  |  |
| GG × ever drinking | 1.00 |  | 1.00 |  | 1.00 |  |
| GT × never drinking | 0.92(0.66-1.29) | 0.64 | 1.21(0.83-1.78) | 0.32 | 0.90(0.70-1.16) | 0.43 |
| TT× never drinking | 1.76(0.75-4.11) | 0.19 | 1.21(0.48-3.05) | 0.70 | 1.38(0.74-2.57) | 0.31 |
| (GT+TT) × never drinking | 0.99(0.72-1.37) | 0.97 | 1.07(0.83-1.38) | 0.60 | 1.01(0.86-1.18) | 0.91 |
| *LEPR* rs6690625 × BMI |  |  |  |  |  |  |
| GG × (BMI≥25kg/m^2^) | 1.00 |  | 1.00 |  | 1.00 |  |
| GT × (BMI<25kg/m^2^) | 0.60(0.40-0.87) | 0.01 | 0.94(0.63-1.40) | 0.76 | 0.74(0.56-0.97) | 0.03 |
| TT × (BMI<25kg/m^2^) | 1.80(0.66-4.87) | 0.25 | 1.00(0.39-2.52) | 0.99 | 1.35(0.69-2.66) | 0.38 |
| (GT+TT) ×(BMI<25kg/m^2^) | 0.66(0.46-0.96) | 0.03 | 0.92(0.72-1.17) | 0.50 | 0.86(0.73-1.01) | 0.06 |

^a^ Adjusted by age, sex, smoking status and alcohol use
